# Evidence-based health information and risk competence

**DOI:** 10.3205/000215

**Published:** 2015-07-09

**Authors:** Ingrid Mühlhauser, Martina Albrecht, Anke Steckelberg

**Affiliations:** 1 Health Sciences and Education, Faculty of Mathematics, Informatics and Natural Sciences, University of Hamburg, Germany

**Keywords:** evidence-based medicine, patient information, consumer information, risk communication

## Abstract

Consumers and patients want to be included in decisions regarding their own health and have an ethically justified claim on informed decisions. Therefore, sound information is required, but health information is often misleading and based on different interests. The risks of disease and the benefits of medical interventions tend to be overestimated, whereas harm is often underestimated. Evidence-based health information has to fulfil certain criteria, for instance, it should be evidence-based, independent, complete, true as well as understandable. The aim of a medical intervention has to be explained. The different therapeutic options including the option not to intervene have to be delineated. The probabilities for success, lack of success and unwanted side effects have to be communicated in a numerical and understandable manner. Patients have the right to reject medical interventions without any sanctions.

## Zusammenfassung

Verbraucher und Patienten wollen in Entscheidungen zu ihrer Gesundheit miteinbezogen werden. Sie haben einen ethisch legitimierten Anspruch auf informierte Entscheidungen. Dazu brauchen sie gute Informationen. Gesundheitsinformationen sind jedoch oft irreführend und interessengeleitet. Krankheitsrisiken und der Nutzen medizinischer Maßnahmen werden üblicherweise überschätzt, der Schaden unterschätzt. Evidenzbasierte Gesundheitsinformationen müssen bestimmte Kriterien erfüllen. Sie müssen wissenschaftsbasiert, unabhängig, vollständig, ehrlich und verständlich sein. Das Ziel einer medizinischen Maßnahme muss erklärt werden. Die möglichen Optionen, einschließlich der Option auf eine Intervention zu verzichten, müssen dargestellt werden. Die Wahrscheinlichkeiten für Erfolg, fehlenden Erfolg und unerwünschte Effekte müssen numerisch und verständlich kommuniziert werden. Medizinische Eingriffe müssen ohne Sanktionen abgelehnt werden können.

## Consumers want to participate in the decision-making process

Patients increasingly want to be included in the medical decision-making process [1]. Surveys among representative sections of the population have shown time and again that, in the event of illness, people want to make decisions together with his or her physician or even independently. Only the minority of people want physicians to decide on their own, and this fact is largely independent of the social or educational background, age or health status [2].

However, health-relevant decisions also concern healthy and symptom-free people in the case of preventive measures, such as life style interventions, health checks, cancer screening tests or measures to reduce risks both in private life as well as at work [3], [4], [5].

We all make decisions on health-related and disease-related topics time and again. In the end, we always decide autonomously whether we accept medical advice or a prescription, take medication as recommended, make use of vaccinations and health check-ups or undergo surgery. The question remains how good is the information as the basis of such decisions? Fact is that consumers and patients want to make ‘informed decisions’, that means, they do not want to be misguided but wish to make good decisions on health prevention or in the event of illness. An informed decision is defined as a decision based on evidence-based knowledge and in accordance with personal preferences [6].

## The lack of evidence-based information

Although an unmanageable amount of information on most topics is nowadays accessible from the internet, only few data correspond to the criteria of high-quality information [7]. Many consumers believe that they make informed and autonomous decisions, although this is not true in many cases [8]. Most people think that they are able to estimate the benefit or risk of medical interventions. In reality, many studies have shown that even physicians, health policymakers and other academics frequently misjudge such benefits or risks [9]. The main reason for misjudgement is misleading information because reliable information is indeed rare [2], [7].

Most information on health issues is incomplete, interest-led and unclear. Risk information, such as in the package leaflets of medicinal products, are often neither understood by consumers nor by physicians, pharmacists or lawyers [10]. Information on cancer screening tests typically results in the considerable overestimation of the benefits and in the underestimation of the risks. Disease risks are often largely overestimated [8]. Overall, patient information results in the disproportionate evaluation of the possible effects of preventive or medical measures [9], [11].

## Is financial counselling better than patient counselling?

Since 2010, banks in Germany are legally bound to provide detailed information on financial transactions. The objectives and preferences of the customers have to be determined followed by the offer of various alternatives and the explanation of the risks involved. The financial advisor has to sign a protocol, and the customer receives a copy. 

However, this procedure is still not necessary in the case of medical interventions. Traditional informed consent forms - for instance, submitted for signature before surgery – mainly serve as a legal safeguard for physicians but not as patient information [12].

Despite the flood of health information available on the internet, consumers have hardly had the chance to participate in medical decision-making in terms of the informed or shared decision-making process that has been advocated for years. Evidence-based patient information (EBPI) in terms of decision aids are lacking as well as structures for the implementation of this concept [2]. 

Various institutions have tried to develop evidence-based health information; in Germany, mainly the Institute for Quality and Efficiency in Health Care (Institut für Qualität und Wirtschaftlichkeit im Gesundheitswesen – IQWiG). However, individual institutions are not able to develop the necessary evidence-based patient information in terms of decision aids for every health topic and particularly not for specific medical decisions. The Institute for Quality and Efficiency in Health Care in Germany in its current form is also unable to provide more than only general health information on most topics.

In the case of specific indications, however, patients want to know their individual risk and the range of medical interventions available in their particular circumstances. Patients, for example, who have been advised to undergo surgery, should have the chance to understand whether the surgical intervention is useful or not. Thus, information on possible surgical interventions including the option of not undergoing surgery (for the time being) should be made readily available. Prognoses with probability details on the benefits and risks of the individual measures over defined periods have to be presented in a clear and understandable manner [13]. To this end, study results including figures should be offered and explained by means of the above-mentioned decision aids, particularly for diseases with uncertain prognosis and complex therapeutic procedures, such as multiple sclerosis or cancer.

So far, such decision aids have only been developed in isolated cases, mostly in the context of research projects. Furthermore, it is difficult for people to adequately check the quality of such decision aids. Quality seals, for instance, the Action Forum on Health Information Systems (Aktionsforum Gesundheitsinformationssystem e. V. – AFGIS) and Health On the Net Foundation (HON), or assessment tools, such as DISCERN, have limited value because their contents are not verifiable with regard to their evidence-based approach [7]. The scientific quality of their contents cannot be assessed without any special knowledge and, in most cases, only with much effort [14]. 

## Criteria for evidence-based health information

For about 20 years, various work groups worldwide have addressed the question of how information on health and disease topics can be presented in such a manner that allows people to make informed decisions [15], [16]. Criteria of information generation and the information process have been defined [13], [15], [16], [17], [18] with regard to content, presentation of the content and the process of information generation. Table 1 (Tab. 1) shows an overview of the most important criteria. In 2008, the Working Group (Section) of Patient Information and Patient Participation of the German Network for Evidence-based Medicine (Fachbereich Patienteninformation und Patientenbeteiligung des Deutschen Netzwerks für Evidenzbasierte Medizin – DNEbM) has already developed minimum standards for information materials on cancer screening [18] and, has compiled criteria for the development and assessment of patient information in a publication entitled “Good Practice of Health Information” (Gute Praxis Gesundheitsinformation) in 2010 [17]. Currently, an updated version is under review (http://www.ebm-netzwerk.de/was-wir-tun/fachbereiche/patienteninformation/gpgi).

Before any medical intervention, patients should be informed on the course of the complaints or disease without any intervention, on the aim of the examination or treatment, on all options as well as on the possible benefits and risks of the individual intervention. The probabilities for success, lack of success and harm have to be presented including figures. The data have to refer to target parameters that are relevant to the patient. Any lack of evidence needs to be disclosed, and data have to be presented in full and unambiguously.

At the University of Hamburg, a working group has been established in cooperation with the Section of Patient Information and Patient Participation of the German Network for Evidence-based Medicine to develop a guideline for the development of patient information [14]. Martina Albrecht and Anke Steckelberg have prepared a manual on the development of health information for the German Federal Institute for Occupational Safety and Health (Bundesanstalt für Arbeitsschutz und Arbeitsmedizin – BAuA) [19]. 

The following examples show what type of information would allow informed decisions (modified according to [20]).

## Description of risks

**An example of disease risk:**

Such information should be differentiated according to sex and age groups. A comparison with other diseases may be useful.

- Risk without any further measures: “Without screening, about 20 in 1,000 women aged between 50 and 60 years will fall ill* with breast cancer and 2 with malignant melanoma within the next 10 years.”
- Risk with further measures: “Despite screening, about 30 in 1,000 women aged between 50 and 60 years will fall ill* with breast cancer and 3 with malignant melanoma within the next 10 years.”

* The correct expression would be “diagnosed with” instead of “fall ill with”. With screening, both breast cancer and skin cancer show persistent increasing incidence rates. Cancer cannot be prevented by screening but it may be diagnosed at an earlier stage. The percentages of overdiagnoses are estimates.

**An example of mortality risk:**

In Germany, about 200 in 1,000 women die of any type of cancer, of whom 34 die of breast cancer and 2 of malignant melanoma.

**An example of communicating benefits:**

For every 1,000 women aged 50 years screened, one less women will die from breast cancer over a period of 10 years. Without screening, this rate would be 4 in 1,000 women; with screening, the rate would be 3 in 1,000 women, which corresponds to a relative risk reduction of 25%.

To what extent, if at all, screening may reduce the mortality rate of melanoma remains speculative. Assessment of the risk-benefit ratio requires randomised controlled studies which are currently non-existing. Without screening, about 2 in 10,000 women aged 50 years will die of malignant melanoma within the next 10 years.

**An example of communicating the incidence of (drug) side effects and risks:**

Information on risks solely by narrative description is deceptive. Not only patients but also physicians, pharmacists and layers overestimate risks in the case of narrative descriptions only [10]. Therefore, the respective EU directive should be followed which stipulates the inclusion not only of statements such as ‘rare’ or ‘common’ but also of the following numerical representation [13]:

- Very common: >10 in 100 (10%)
- Common: 1 to 10 in 100 (1% to 10%)
- Uncommon: 1 in 1,000 to 1 in 100 (0.1% to 1%)
- Rare: 1 in 10,000 to 1 in 1,000 (0.01% to 0.1%)
- Very rare: <1 in 10,000 (0.01%)

Comprehensive descriptions on the criteria for evidence-based patient information are to be found in other publications [8], [9], [11], [13], [14], [17], [18], [19], [20].

## Proposals on enhancing risk competence in the context of patient and consumer information

Consumers and patients increasingly want to participate in decisions on health and disease issues. They are entitled and have an ethically justified claim to informed participation in the medical decision-making process. Such participation requires adequate information and decision aids, which are only available as an exception. Most information is misleading, interest-led and incomplete, and there is a lack of structures for the sustainable, comprehensive provision of evidence-based information.

Generally, society is known for its innumeracy. Even physicians, pharmacists, lawyers and other academics are often unable to understand risks. Such *health illiteracy* provides a fertile base for misuse and manipulation. Evidence-based information is marked by the evidence of its contents as well as by the form of its presentation. Before a medical intervention, patients should be informed on the course of the complaints or disease without any intervention, on the aim of the examination or treatment, on all options as well as on the possible benefits and risks of the individual intervention. The probabilities for success, lack of success and unwanted side effects have to be communicated in a numerical manner. The data have to refer to target parameters that are relevant to the patient. Any lack of evidence needs to be disclosed, and data have to be presented in full and unambiguously.

Extensive studies available by now show that knowledge and understanding are significantly influenced by the manner in which information is presented. Information which does not meet scientific quality criteria leads to substantial misconceptions and poor decisions. Consumers or patients are just as much affected as decisions on health policies or health economy.

Most medical decisions are made under uncertainty. Patients must be involved in the decision-making process, particularly in the case of chronic diseases with unpredictable courses, a variety of different treatment options and a variable benefit-risk ratio. Patients should also be allowed to make an informed decision in the case of a surgical intervention. Traditional informed consent forms are insufficient in this respect because they rather serve as a legal safeguard for physicians but not as balanced information for patients.

Patients, who have been advised to undergo surgery, should have the chance to understand whether the surgical intervention is useful and necessary or not. Thus, information on possible surgical interventions including the option of not undergoing surgery (for the time being) should be made readily available. Prognoses with probability details on the benefits and risks of the individual measures over defined periods have to be presented in a clear and understandable manner [13]. To this end, study results including figures should be offered and explained by means of the above-mentioned decision aids, particularly for diseases with uncertain prognosis and complex therapeutic procedures, such as multiple sclerosis or cancer.

Communication regarding the frequencies of (drug) side effects and risks in the package leaflets of medicinal products has to be designed in such a way that allows adequate risk assessment. If feasible, data on comparative groups (placebo or standard therapies) should also be provided. Because the currently used form leads to misconceptions, risk comprehension is not guaranteed.

Any health education must be based on the methods of evidence-based medicine, and this principle should apply to physicians as well as to health carers and teachers in health schools. Curricula are available and have become an integral part of health education in other countries. For patient or consumer representatives and health or patient counsellor, already completed pilot projects are now available in which 5-day courses (or courses of similar formats) on critical health literacy are evaluated.

The need for information and participation is high in all sections of society, independent of age, education and health status. However, according to new research, about 14% of adults in Germany are assumed to be unable to read and write. Presentation formats of health information have to acknowledge this fact as well as the different requirements of other vulnerable groups.

## Notes

### Competing interests

The authors declare that they have no competing interests.

### Remarks to this article

Parts of this article on the topic “Enhancing risk competence” have been published in similar or identical form, for instance in: Mühlhauser I, Albrecht M, Steckelberg A. Evidenzbasierte Gesundheitsinformation. Zentralbl Arbeitsmed Arbeitsschutz Ergonomie. 2014;64(5):334-7. DOI 10.1007/s40664-014-0054-0 [21]).

## Figures and Tables

**Table 1:**
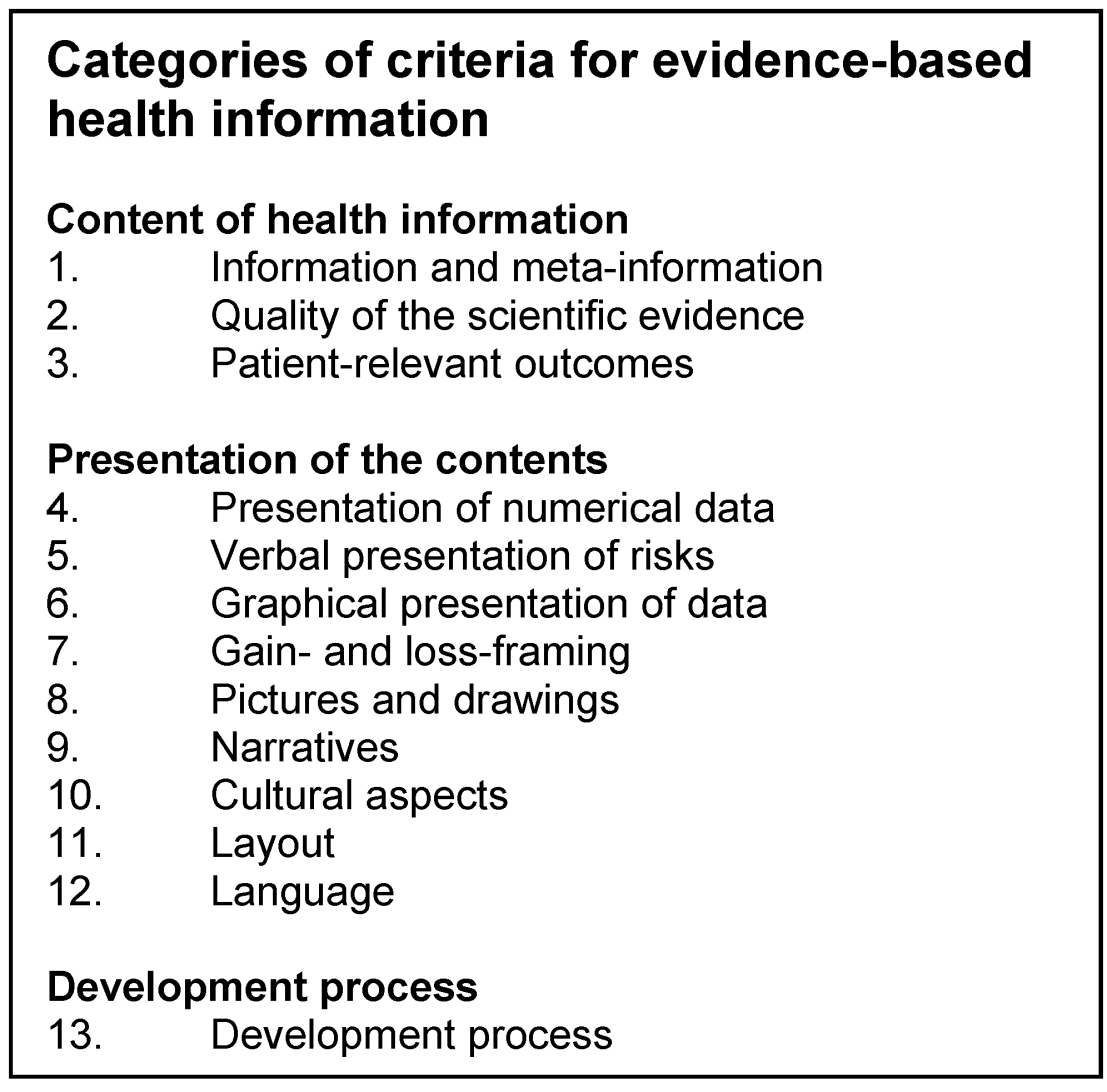
Categories of criteria for evidence-based health information Adapted from: Bunge M, Mühlhauser I, Steckelberg A. What constitutes evidence-based patient information? Overview of discussed criteria. Patient Educ Couns. 2010 Mar;78(3):316-28. DOI: 10.1016/j.pec.2009.10.029 [13].
